# Stakeholder Network Analysis for Front-of-Pack Labeling in China

**DOI:** 10.3389/fnut.2022.871062

**Published:** 2022-05-23

**Authors:** Xuejun Yin, Lihong Ye, Xin Xin, Lin Xiang, Yue Yu, Ruijie Yan, Kehan Wen, Maoyi Tian, Alexandra Jones, Simone Pettigrew, Juan Zhang

**Affiliations:** ^1^School of Population Medicine and Public Health, Chinese Academy of Medical Sciences and Peking Union Medical College, Beijing, China; ^2^The George Institute for Global Health, University of New South Wales, Newtown, NSW, Australia; ^3^Faculty of Psychology, Beijing Normal University, Beijing, China; ^4^School of Public Health, Harbin Medical University, Harbin, China

**Keywords:** front-of-package labeling, Net-map, stakeholder mapping, stakeholder analysis, network analysis, China

## Abstract

**Background:**

Front-of-pack (FoP) labeling on packaged foods is recommended by the World Health Organization (WHO) to reduce diet-related non-communicable diseases, but it has not yet been implemented in China. The introduction of FoP labeling is driven by multiple institutions and stakeholders. This study aimed to identify key institutional actors involved in FoP labeling and describe links between actors to support future FoP labeling policies and programmes in China.

**Methods:**

The Net-Map method was adopted. We conducted Net-map activities with eight participants using face-to-face interviews between November 2020 and May 2021. Participants were asked to identify actors involved in the development and implementation of FoP labeling in China, describe networks among actors according to pre-defined link types (command, dissemination, funding, and technical assistance), and estimate influence of each actor within the FoP labeling landscape. Social network analysis measures of cohesion and centrality were used to describe each network. Gephi software was used for social network analysis and network visualization.

**Results:**

Forty-one unique actors were identified across seven actor categories including government agencies (*n* = 14), technical support agencies (*n* = 7), professional associations (*n* = 10), food industry groups (*n* = 2), media groups (*n* = 4), international organizations (*n* = 3), and a consumer group (*n* = 1). Weighted influence scores among actors ranged from 0.13 to 3.13. The Department of Food Safety Standards, Risk Surveillance and Assessment of the National Health Commission (DFSSRSA of NHC) was the actor with the highest weighted influence score, followed by the Bureau of Disease Prevention and Control of National Health Commission, Chinese Nutrition Society, and the National Institute for Nutrition and Health of Chinese Center for Disease Control and Prevention. DFSSRSA of NHC played a central role in both command and technical assistance networks. State-owned media had the greatest betweenness and outdegree centrality in the dissemination network. The Chinese Nutrition Society was in the central position and provided funding to diverse actors in the funding network.

**Conclusions:**

A variety of multisectoral actors have an interest in the introduction of FoP labeling policies and programmes. Effectively engaging key actors identified in this study can generate a multisectoral commitment to advance FoP labeling policies and programmes in China.

## Introduction

Non-communicable diseases (NCDs) are the leading cause of death and disability globally (1, 2). Suboptimal diet is a major contributor to the surging global burden of obesity and diet-related NCDs (3, 4). The World Health Organization (WHO) recommends implementing front-of-package (FoP) labeling on packaged food as a “Best Buy” intervention to address the growing global burden of diet-related NCDs (5). FoP labeling can support consumers to make informed and healthier food choices, and encourage industry to reformulate food products to remove health-harming ingredients (6). Worldwide, FoP labeling schemes were reported by 55 countries, of which 37 provided detailed information available (7).

Over recent decades, dietary patterns have changed significantly in China, characterized by increased consumption of foods high in energy, total fats, sugars or sodium, and decreased intake of fruit, vegetables, and dietary fiber (8). Population consumption of sodium and saturated fat far exceeds recommended daily intake levels (9). Unhealthy diets are associated with increased prevalence of hypertension, cardiovascular diseases, and diabetes (10, 11). To address this challenge, the Chinese Government has released the Healthy China Movement to prioritize the promotion of healthy diets with the goal of cutting dietary salt, saturated fat, and sugar intake nationwide by 2030. In addition, it encourages the food industry to provide supplemental nutrition information on the front of packages to help consumers identify healthier food options (12).

Although there has been increased interest in establishing FoP labeling as a nutrition policy priority, such labeling has not been implemented in China. A recent qualitative study of barriers and facilitators to developing a feasible and acceptable FoP labeling policy in the Chinese context indicated that lack of planning and engagement with stakeholders were some of the barriers to introducing FoP labeling in China (13). Considering the multisectoral nature of food governance, the development of FoP labeling involves diverse stakeholders from health, industry, regulatory, and trade sectors (14). This makes it important to understand how different stakeholders could influence the introduction and implementation of FoP labeling in China.

Stakeholder network analysis has been successfully used to inform explicit strategies to engage diverse stakeholders in the formation and implementation of policies across diverse fields (15). This study adopted stakeholder network analysis to identify the roles of stakeholders in the introduction and implementation of FoP labeling in China, describe the relationship between stakeholders, and understand their influence on the potential introduction and implementation of FoP labeling.

## Materials and Methods

### Study Design

This study was carried out using Net-Map, a methodology that facilitates visualization of stakeholder's interactions and the way these interactions influence the policy decision-making process (15). It combines stakeholder analysis, social network analysis, and power mapping, and has previously been used to identify stakeholders, examine their interactions, and rank their influence on policy development and implementation processes for infant and young child feeding policies and programs in Asia (16–18). The study received ethics approval from the Chinese Centre for Disease Control and Prevention (Approval No.: 202024).

### Identification of Study Participants

An initial participant list was generated from the qualitative study that we conducted to identify influential factors of FoP labeling development in the Chinese context (19). During qualitative interviews, stakeholders were named if they have an influence on the implementation of FoP labeling in China. Then, we purposively selected those who were knowledgeable about FoP labeling policy processes and have been involved in the development and implementation of existing food policies in China through research team group discussion. Participants were sent research written invitations by the senior investigator (JZ) explaining the purpose of the activity and requesting their participation. Research information and an informed consent form were provided to those who expressed their interest in participation. The participants were given sufficient time to read the information before commencing data collection. Eight participants agreed to take the Net-map activities (Table 1).

**Table 1:** Characteristics of participants.

**Participant**	***N***
	**(Total = 8)**
**Organization**
National health commission	1
Chinese center for disease control and prevention	3
Beijing centers for disease prevention and control	1
China national center for food safety risk assessment	1
Center for international communication studies of Tsinghua university	1
Chinese nutrition society	1
Number of female/male participant	5 / 3
**Years of experience**
5–10 years	1
10–15 years	4
15+ years	3

### Data Collection

Data were collected through individual interviews from November 2020 to May 2021. The process of data collection consisted of three parts: (1) stakeholder mapping to identify institutional actors who influence FoP labeling policy at the national level; (2) social network analysis to understand the direct links between actors in terms of command, dissemination, funding, advocacy, and technical assistance; and (3) power mapping to estimate the influence of these actors on progressing the development of FoP labeling (20).

The research team initially prepared a list of potential institutional actors to be discussed for stakeholder mapping. The list was formed from the 2020 China Nutrition 30 Forum list of members of the Advisory Board of State Nutrition Plan (2017-2030) (21), and stakeholders who participated in the amendment of nutrition labeling standards (22). Potential actors were categorized into government (i.e., National Health Commission, State Administration for Market Regulation), technical support agencies (i.e., academic organizations, Center for Disease Control and Prevention at national level, and provincial level), professional associations related to food and nutrition (i.e., Chinese Nutrition Society, China Consumers Association), the food industry, and the media groups. The operational definition of actor groups was shown in Table 2. During the interviews, additional actors mentioned by participants but not in the prepared list were added to the list of actors.

**Table 2:** Definition of actor groups.

**Actor group**	**Operational definition**	**Examples of organization**
Government	The chief administrative authority in China is the State Council. Government agencies are ministries that are under the State Council and centrally administered government organizations that report directly to the state council.	National Health Commission, State Administration for Market Regulation
Technical support agencies	Entity established or controlled by the government, including public institutions, institutions of higher education and related research institutions.	Chinese Centre for Disease Control and Prevention, China National Centre for Food Safety Risk Assessment, George Institute for Global Health
Associations	Association refers to a group or organization voluntarily formed by individuals and single organizations to achieve a certain goal by signing an agreement.	China Consumers Association, Chinese Nutrition Society, China Cuisine Association
Media	Media refers to a person or entity engaged in disseminating information to the general public, including state-owned media, newspaper and magazines, internet media and We-media.	Xinhua News Agency China food newspaper We Media_Gu Zhongyi
International organization	Multilateralism and international governmental organizations are established by a treaty or be an instrument governed by international law and possessing its own legal personality.	World Health Organisation, United Nations International Children's Emergency Fund Food and Agriculture Organization of the United Nations
Food industry	Food manufacturers producing packaged food. They were divided into three types: (1) multinational food company with business activities in more than one country, (2) large domestic food company with more than 300 employees or an annual income of more than 200 million, (3) domestic middle and small food company with <300 employees and an annual income of <200 million.	Nestle (multinational food company) PANPAN foods (large domestic food company)

Once all actors had been identified, participants were asked to place stick-on notes representing each actor on a large sheet and link the actors to each other. The interviewer explained the definition of each link type and instructed participants to connect the actors with colored arrows according to pre-defined links (command, dissemination, funding, or technical assistance). The pre-defined link types and their definitions were listed in Table 3. Links were displayed as arrows between two actors, indicating the direction of the link. For example, a funding link going from actor A to actor B indicated that actor A provides funding/financial incentives to actor B. If there is a mutual exchange, the arrow has two heads. If there is an exchange within the actor, the arrow directs to itself. The links reflected the existing relationships at the point of interview instead of those that should or will exist in the future. The interviewer guided the participant to finish one kind of link before starting another. The final network map in each interview was explained and critically reflected by the participant.

**Table 3:** Description of links assessed in the Net-map interviews.

**Link type**	**Operational definition**
Funding (F)	Any two or more actors/institutions are linked by giving or receiving money or financial incentives (for example, if actor A funds project to actor B, then a funding link goes from A to B).
Command (C)	Any two or more actors/institutions are linked by giving or receiving commands/directives (for example, if actor A tells actor B that an activity must be implemented, then a command link goes from actor A to actor B).
Technical Assistance (TA)	Any two or more actors/institutions are linked by giving or receiving technical assistance (for example, if actor A offers advice/skilled-based training to actor B, then a technical assistance link goes from actor A to actor B).
Dissemination (D)	Any two or more actors/institutions are linked by dissemination of information (for example, if actor A spreads any developed information to Actor B, then a dissemination link goes from actor A to actor B).

Finally, participants were asked how much influence each actor had with respect to developing or implementing FoP labeling policies and programs in China. The participants ranked each actor with an influence value from 0 to 5. The more influence an actor was perceived to have, the higher the influence score. According to the answers, influence scores were put next to the actor cards.

Data collected at each interview included photographs of the maps, handwritten notes, and an audio recording. All interviews were conducted by a trained interviewer (LY/XX) with two or three support note-takers (LX, YY, RY, KW) at the interviewee's office or private space. Written consent was obtained from each participant before engaging in the Net-map activities.

### Data Analysis

Data from the photographed maps of the actors, links, and influence scores from each interview were entered into an Excel file. Data on actors and links across the eight interviews were merged in one dataset and imported into Gephi for analysis. Each actor's relative influence was weighted by the number of nominations across the eight interviews. In a visual illustration of a social network, actors were presented by nodes and links represented by lines and arrows. The actors were also color-coded by actor groups and sized proportionately to the level of weighted relative influence. The thickness of lines represented the number of times links were mentioned. Actor networks were mapped for each link type (i.e., command, dissemination, funding, technical assistance). Social network analysis measures of cohesion (density and distance) and measures of centrality (in-degree, out-degree, as well as betweenness degrees), as defined in Table 4, were used to describe each network (20).

**Table 4:** Social network metrics.

**Metrics**	**Interpretation**	**Definition**
**Size reflects the distribution of actor groups**		
Number of actors (nodes)	Size of the network	Number of actors in the network
Number of links	How “busy” the network is in total	Number of connections between actors in the network (in total)
**Cohesion reflects the interconnectedness of actors in a network**		
Average distance	The proximity of nodes to one another	The average number of links between nodes. Where distances are great, it may take a long time for information to diffuse across a network; moreover, actors who are closer to more others may be able to exert more power than those who are more distant
Density	The extent to which nodes are interconnected	The proportion of all links that are present out of all possible links. Density is a ratio that can range from 0 to 1; the closer to 1 the density is, the more interconnected the network is
**Centrality reflects the prominent actors within a network**		
In-degree	Quantifies the inputs, or directions, received by an actor from the other actors in the network	Measures the number of links directed at an actor, representing the input received from a particular network
Out-degree	Quantifies the links of an actor to other actors in the network	Measures the number of links from an actor directed to other actors in the network, representing the input provided to a particular network
Betweenness	Represents the control an actor has over the flow of inputs across a network	Measures the number of times an actor lies on the shortest path between two other actors within a network, representing the control an actor has over the flow of inputs across a network

## Results

### Characteristics of Actors

**Figure 1:**
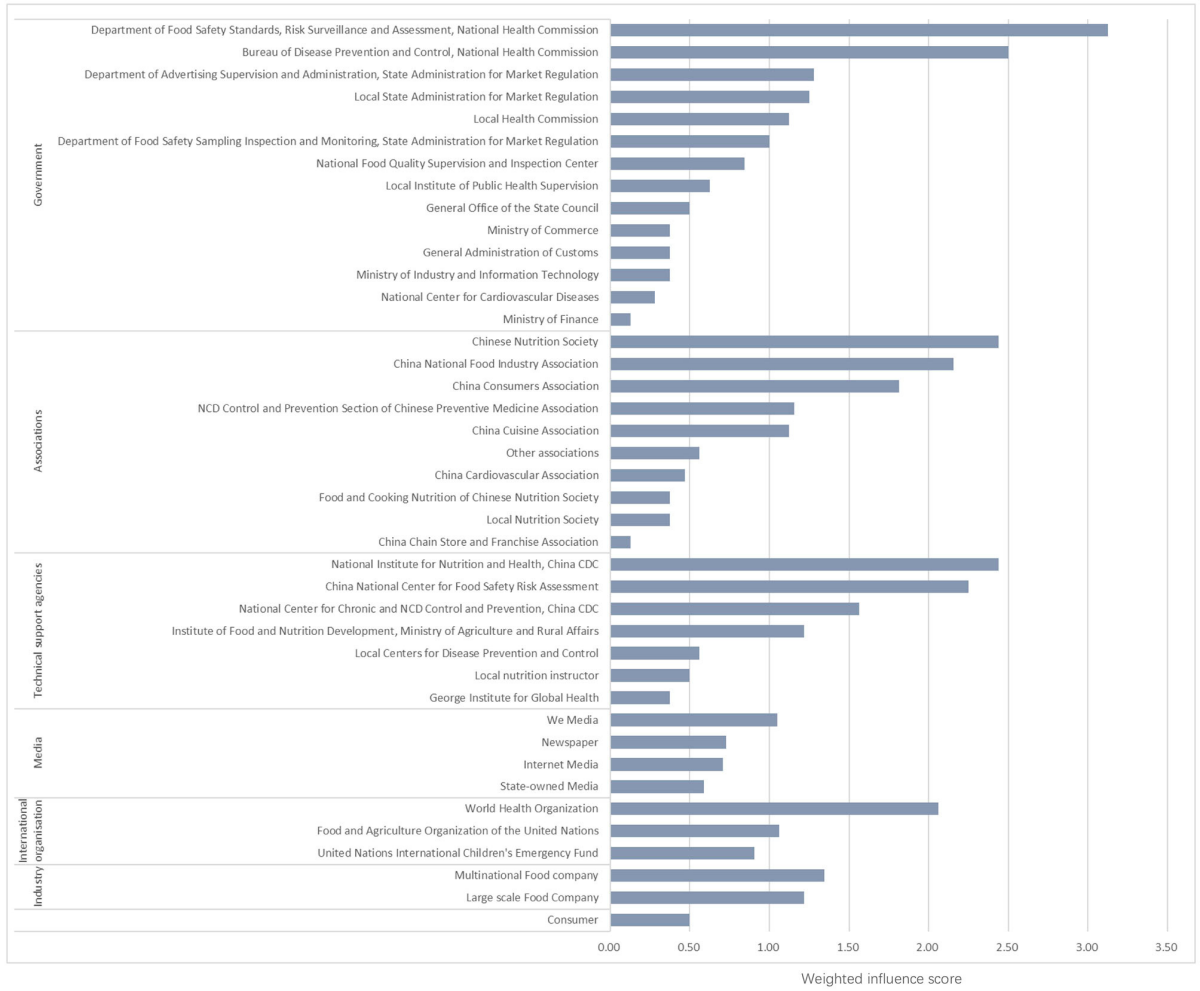
Relative influence of actors.

From the eight participants interviewed, 41 actors were identified. These were categorized into seven actor groups: government agencies (*n* = 14), technical support agencies (*n* = 7), professional associations (*n* = 10), food industry groups (*n* = 2), media groups (*n* = 4), international organizations (*n* = 3) and a consumer group (*n* = 1). The most frequently named actors were mentioned five times across the eight participant interviews. The specific actors were: the Bureau of Disease Prevention and Control of National Health Commission (BDPC of NHC); the Department of Food Safety Standards, Risk Surveillance and Assessment of National Health Commission (DFSSRSA of NHC); China National Food Industry Association (CNFIA); China Consumers Association (CCA2); Chinese Nutrition Society (CNS); China National Center for Food Safety Risk Assessment (CFSA); National Center for Chronic and Non-communicable Disease Control and Prevention of Chinese Center for Disease Control and Prevention (NCNCD of CCDC); National Institute for Nutrition and Health of Chinese Center for Disease Control and Prevention (NINH of CCDC); and World Health Organization (WHO). The weighted influence score among actors ranged from 0.13 to 3.13 (Figure 1). DFSSRSA of NHC and BDPC of NHC had the highest influence scores, followed by CNS and NINH of CCDC.

### Command Network

**Figure 2:**
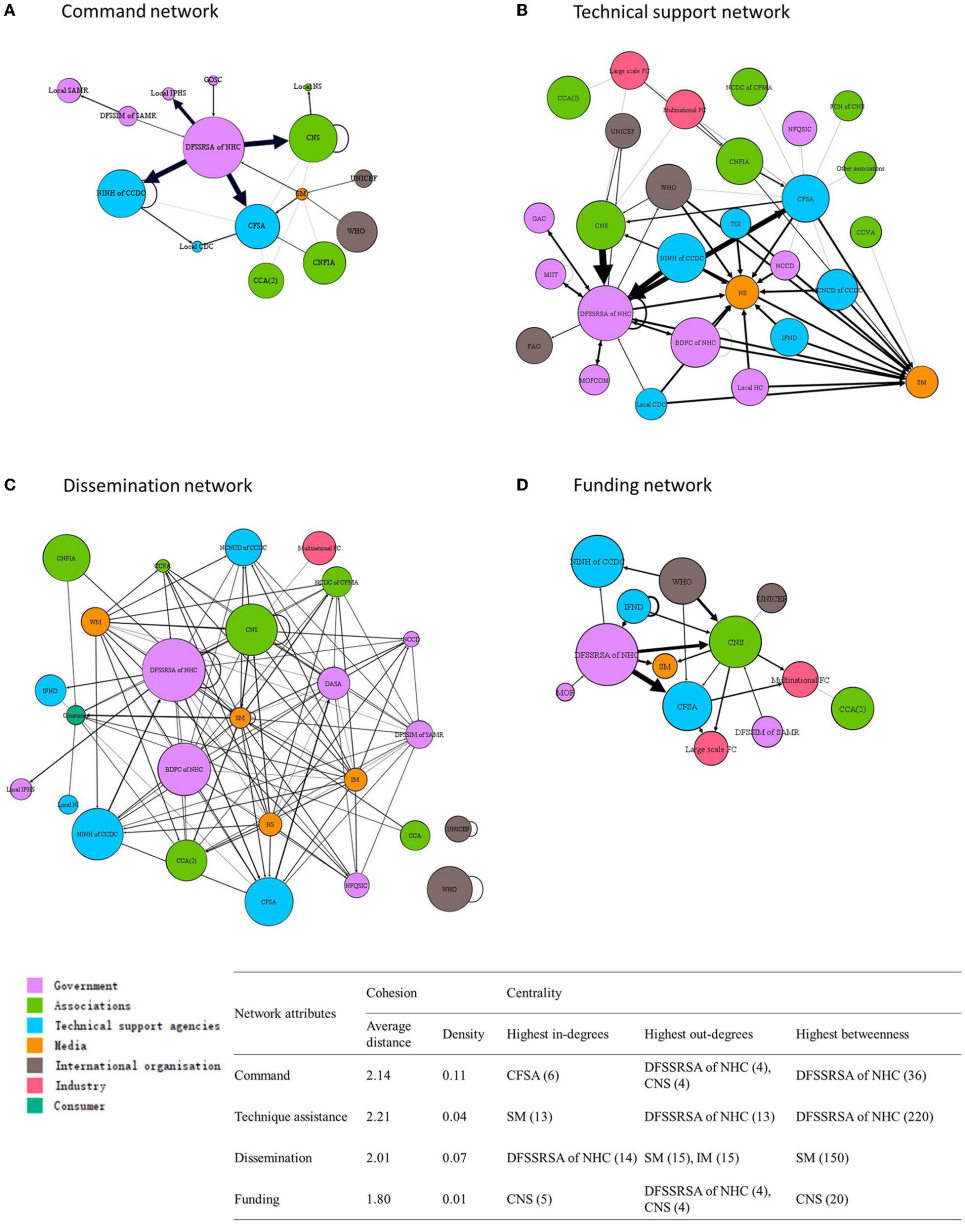
Networks of actors for FoP labeling policy and programming in China regarding to **(A)** command, **(B)** technical support, **(C)** dissemination, and **(D)** funding. Nodes are sized by weighted average influence and colored by actor groups. Width of lines between nodes indicates the strength of links.

The command network was comprised of 15 actors connected by 30 unique links (Figure 2A). Actors in this network were from government agencies (*n* = 5), professional associations (*n* = 4), technical support agencies (*n* = 3), international organizations (*n* = 2), and the media (*n* = 1). The network density was 0.11, indicating 11% of total possible links across the command network had been achieved. The mean of the shortest path lengths among all connected actors in the command network was 2.14. The DFSSRSA of NHC was a central actor in the command network, with the greatest betweenness centrality, indicating that the DFSSRSA of NHC was the most influential over the flow of command between actors by its position in the network. DFSSRSA of NHC from the government group, CNS from the professional association group (out-degree centrality, *n* = 4 links), and NINH of CCDC from the technical support agencies (out-degree centrality, *n* = 3 links) were described as regularly providing commands to other actors in the network. CFSA received commands from the largest number of other actors (in-degree centrality, *n* = 6 links). The food industry and consumers were not mentioned in the command network.

### Technical Assistance Network

The technical assistance network was comprised of 28 actors connected by 71 unique links (Figure 2B). Actors in this network were from government agencies (*n* = 8), professional associations (*n* = 7), technical support agencies (*n* = 6), international organizations (*n* = 3), food industry (*n* = 2), and the media (*n* = 2). The density of the technical assistance network was only 0.04 (only 4% of possible links across technical assistance network had been achieved) and the mean of links to provide technical assistance among connected actors was 2.21. The DFSSRSA of NHC was the central actor with the greatest betweenness centrality. It was also the most common actor providing technical assistance to other actors (out-degree centrality, *n* = 13 links). Other actors often providing technical assistance included NINH CCDC, WHO, BDPC of NHC, CFSA and domestic large food companies (out-degree centrality, all *n* = 4 links). Media groups, such as the state-owned media, received the greatest support from other actor groups (in-degree centrality, *n* = 13 links).

### Dissemination Network

The dissemination network was comprised of 26 actors connected by 125 unique links (Figure 2C). These links encompassed all seven actor groups: government agencies (*n* = 7), professional associations (*n* = 5), technical support agencies (*n* = 5), international organizations (*n* = 2), the media (*n* = 5), industry (*n* = 1) and consumer (*n* = 1). The network density indicated only 7% of possible links across the network had been achieved. The average distance between any two actors was 2.95. The official media had the highest betweenness centrality, meaning it had more control over the flow of information and acted as a key bridge within the network. Internet media (out-degree centrality, 15 links), state-owned media (out-degree centrality, 15 links), newspaper (out-degree centrality, 14 links), and We media (out-degree centrality, *n* = 14 links) were cited as being influential for their role in information dissemination. DFSSRSA of NHC was mentioned as having the highest tendency to receive information from the other network actors (in-degree centrality, *n* = 14 links), followed by Department of Food Safety Sampling Inspection and Monitoring, State Administration for Market Regulation (DFSSIM of SAMR), Bureau of Disease Prevention and Control (BDPC), and Department of Advertising Supervision and Administration, State Administration for Market Regulation (DASA) (in-degree centrality, all *n* = 13 links).

### Funding Network

The funding network was comprised of 13 actors connected by 24 unique links (Figure 2D). Actors in the funding network were from government agencies (*n* = 3), professional associations (*n* = 2), technical support agencies (*n* = 3), international organizations (*n* = 2), the food industry (*n* = 2), and the media (*n* = 1). The funding network density was 0.01, indicating that actors within funding network were generally not very interconnected with each other. The average distance between any two actors was 1.8 links. The CNS was described as playing an important role in passing on funds, resulting in the highest betweenness centrality in the network. The CNS also had the highest in-degree centrality, receiving funding from other government and international organization actors (in-degree, 5 links). DFSSRSA of NHC and CNS provided direct funding support to the greatest number of other actors (out-degree, 15 links).

## Discussion

This study identified a range of multisectoral actors who can be engaged in the introduction and implementation of FoP labeling policy. It provides a visual snapshot of how these actors are linked in different ways (command, technical assistance, dissemination, and funding networks) at the formative stage that will be important to generating momentum toward implementing this policy. More actors were connected in technical assistance and dissemination networks than in the command and funding networks. The low density of networks indicated a lack of interconnection across actors in networks. Therefore, actors in central positions (i.e., DFSSRSA of NHC in both command and technical assistant networks, the Official media in the dissemination network and the CNS in the funding network) have a high impact on the flow of information and resources.

Influence scores from the Net-map analysis indicated that government agencies have the highest influence, followed by technical support agencies and professional associations. A formal government-led stakeholder engagement process should be used to facilitate a collaborative approach to the development of a feasible and acceptable FoP labeling system in China. National Health Commission and State Administration for Market Regulation were suggested to lead the process considering several departments in those institutes having high influence and central positions in different networks. The leading role of government found in our study is in line with successful experience in developing FoP labeling programs from other countries. For example, in France and Israel, FoP system development has been led by the governments, with stakeholder input managed with government oversight (23–25). In France, the FoP labeling was recommended by the Ministry of Health, the Ministry of Agriculture and Food and the Ministry for the Economy and Finance. The legislation process of FoP labeling was discussed with the food manufacturing, retailing industry, scientists, and consumers (25). The Israeli Government initiated the Healthy Israel 2020, including legislative and regulatory proposals for nutrition labeling. The government established a regulatory Committee consisting of representatives from government ministries, academia, civil society organizations and the food industry to work out recommendations for FoP labeling development (23). A report on FoP labeling systems around the world also identified government-led systems as best practice (14).

DFSSRSA of NHC was identified as the most influential actor in the command, technical assistance, and funding networks. It occupied critical gatekeeping positions and would have an important role in sharing information, knowledge, and resources between actors. With the highest out-degree in the command network, DFSSRSA of NHC was described as possessing the most decision-making power on FoP labeling policy. DFSSRSA of NHC is responsible for organizing the drafting of food safety standards, carrying out monitoring, evaluation of food safety risks, and undertaking reviews of the safety of new food ingredients, food additives, and other food-related products (26). It has been responsible for the implementation of the National Nutrition Program (NNP), which has been the influential national nutrition policy aiming to improve nutrition regulatory system and population nutrition status through strengthening the nutritional capacity within all levels and sectors, providing proper guidance to food consumption behavior, dietary patterns, and healthy lifestyle across the lifecycle (27). The findings of the present study support the importance of engaging the DFSSRSA of NHC as a critical stakeholder during the implementation of FoP labeling.

In spite of the leading role of government in each of the specified Net-map networks, all networks also included a diverse spread of actor groups. Results of this study indicate the development of FoP labeling would require collaboration among government, technical support agencies, professional associations, media, and consumers. NINH of CCDC was described as playing an important role in providing commands to other technical support agencies (local CDC and CFSA). CFSA was reported to exchange technical support with governments, professional associations, international organisations, and the food industry, which is in line with its role of providing important technical support in standard development and regulatory supervision (28). CNS was identified as a highly influential professional association, providing commands to local nutrition societies. Unlike DFSSRSA of NHC, whose connections were mostly with government and technical support agencies, CNS was found to engage with all actor groups, in particular with international organizations, the food industry, and consumer groups. Therefore, CNS may play an important role in fostering collaborations among diverse actors to advance FoP labeling policies and support the dissemination of information.

Notably, multinational food companies and domestic large food companies were mentioned in the technical support and funding networks. Collaborations should account for declarations and management of conflicts of interest to ensure that evidence-based policy is developed. The WHO has developed a draft tool for safeguarding conflicts of interest in developing national nutrition policies and the Pan American Health Organization (PAHO) has developed a roadmap for how countries can apply this in practice (29, 30). Those documents can be trialed in China to help identify, prevent, and manage potential conflicts of interest in potential engagement with non-State actors in the policy development and implementation of FoP labeling at Country Level. Although WHO was regarded as an actor of influence in the process of planning a FoP labeling scheme, the results of this study indicate that it has not been highly involved in existing networks. Connections were limited to providing funding to CNS, CFSA, and CDC, and technical assistance to media groups. To date, the involvement of consumers appears to have been minimal in all networks. As the goal of FoP labeling is to help consumers make healthier food choices, the adequate participation of consumers and consumer groups to represent these interests should be considered to increase the acceptability and optimize the benefits of FoP labeling program.

While appropriate engagement with stakeholders has been identified as an important component of developing and implementing FoP labeling policy, the current study appears to be the first to use the Net-map method to systematically assess how actors interact with and influence each other in the current Chinese context. The structure of actors influences the success or failure of policies. Stakeholder social network analysis is increasingly used to understand complex process and interactions among actors. The Net-map method increases network understanding by integrating the drawing of multiplex social networks into their impact pathway. This method adopts a participatory strategy. Any uncertainty can be discussed throughout the mapping process so that the probability of misunderstanding is low. The nature of the relationships identified in this study can inform future research and advocacy to strengthen stakeholder engagement and FoP labeling implementation in China. Our study had several limitations. First, collecting data through individual interviews may lead to less precision and validity of information recall compared to applying the Net-Map approach in group interviews. However, Net-Map interviews with individuals have been previously reported and are considered valid (15, 16, 18). Data quality was optimized by systematic planning and implementation of data collection, training of interviewers, and providing clear definitions of terminology used in the Net-map activities. Second, this study is cross-sectional and therefore was unable to capture the dynamic nature of relationships between actors. Third, data were collected from only eight interviews. The sample size was similar to studies using the same methods for breastfeeding policies in Ghana and intersectoral cooperation in health promotion in Southern Germany (16, 31). At the same time, Net-map activities involve subjective experiences that may influence the perception of networks. We reduced sampling bias and perception bias by including participants who had high levels of knowledge and expertise in this area and they were from diverse actor groups.

## Conclusions

The development and implementation of FoP labeling in China will require government leadership and effective multisectoral collaboration. The results of this study indicate that Department of Food Safety Standards, Risk Surveillance and Assessment, National Health Commission is likely to have the strongest influence on progressing the introduction and implementation of FoP labeling and occupies a critical gatekeeping position in different networks. The apparent low density of networks suggests that increased connection among stakeholders could improve the process of developing FoP labeling.

## Data Availability Statement

The original contributions presented in the study are included in the article/supplementary materials, further inquiries can be directed to the corresponding authors.

## Ethics Statement

The study received ethics approval from the Chinese Centre for Disease Control and Prevention (Approval No.: 202024). The patients/participants provided their written informed consent to participate in this study.

## Author Contributions

JZ obtained funding and designed the study. XY and LY wrote the first draft and analyzed the data. XX, YY, KW, and RY developed the data collection tools. LY, LX, RY, and XX collected data. MT, AJ, SP, and JZ contributed to interpretation of the results. All authors have reviewed and provided feedback/edits on drafts of the manuscript and approved the final version for submission.

## Funding

This study was supported by World Health Organization Representative Office in China (Reference no.: 20200608SR).

## Conflict of Interest

The authors declare that the research was conducted in the absence of any commercial or financial relationships that could be construed as a potential conflict of interest.

## Publisher's Note

All claims expressed in this article are solely those of the authors and do not necessarily represent those of their affiliated organizations, or those of the publisher, the editors and the reviewers. Any product that may be evaluated in this article, or claim that may be made by its manufacturer, is not guaranteed or endorsed by the publisher.
